# Supplementary material for: Synthesis of Novel Kaolin-Supported g-C3N4/CeO2 Composites with Enhanced Photocatalytic Removal of Ciprofloxacin
Source: Materials (Basel). 2020 Aug 28;13(17):3811. doi: 10.3390/ma13173811 (PMC7503662; doi:10.3390/ma13173811)
Supplement: Supplementary file 1 [file materials-13-03811-s001.pdf]

materials

Supplementary Materials

Synthesis of Novel Kaolin-Supported g-C_3N_4/CeO_2 Composites with Enhanced Photocatalytic Removal of Ciprofloxacin

Preparation of Materials

Preparation of Kaolin/CeO_2 Composite

The detailed synthesis process of kaolin/CeO_2 as follows kaolin/CeO_2 composite was prepared. 1.305 g of $Ce(NO_3)_3·6H_2O$ and 14.400 g of NaOH were dissolved in 60 mL distilled water under magnetic stirring for 2 h. Then, the homogeneous solution was transferred to a 100 mL Teflon-lined stainless steels autoclave and heated at 180 °C for 24 h. Subsequently, the autoclave was cooled to room temperature naturally. The resulted precipitate was washed three times with distilled water and ethanol. Then, the resulting product is added to 60 mL of deionized water and stirred with magnetic force for two hours to form suspended solution A. Kaolin (1.0 g) was immersed in 30 mL of ethanol, magnetically stirred for 30 min and then sonicated for 2 h, marked solution B. Afterwards the suspended solution A was slowly added to the suspension B, stirring for 12 h. The obtained product was dried in an oven at 80 °C for 12 h and heated at 450 °C (heating rate of 5 °C /min) for 3 h in a muffle furnace. The obtained sample was kaolin/CeO_2.

Preparation of CeO_2/g-C_3N_4 Composite

CeO_2/g-C_3N_4 composite was prepared, 1.305 g of $Ce(NO_3)_3·6H_2O$ and 14.400 g of NaOH were dissolved in 60 mL distilled water under magnetic stirring for 2 h. Then, the homogeneous solution was transferred to a 100 mL Teflon-lined stainless steels autoclave and heated at 180 °C for 24 h. Subsequently, the autoclave was cooled to room temperature naturally. The resulted precipitate was collected, washed three times with distilled water and ethanol. Then, taking a certain the result product is added to 60 mL of deionized water and stirred with magnetic force for two hours to form suspended solution A. Then, a certain amount of g-C_3N_4 was immersed in 30 mL of ethanol, magnetically stirred for 30 min and then sonicated for 2 h. The resulting suspended solution B. Afterwards the suspended solution A was slowly added to the suspension B, under stirring which was continued for 12 h. The obtained product was dried in an oven at 80 °C for 12 h. Then, the solid was ground into powder and heated at 450 °C (heating rate of 5 °C /min) for 3 h in a muffle furnace. The obtained solid was CeO_2/g-C_3N_4.

Preparation of Kaolin /g-C_3N_4 Composite

Kaolin/g-C_3N_4 composite was prepared, A certain amount of the kaolin was dispersed in an ethanol solution and stirred for 12 h (solution A). A certain amount of g-C_3N_4 powder was dispersed in an ethanol solution and stirred for at least 8 h (solution B). Then, solution B was added dropwise to the solution A and stirred continuously for 1 h (solution C). The solution C was continuously stirred for 24 h at room temperature. The obtained suspension was centrifuged and the obtained solid dried in an oven at 60 °C for 12 h. The dried sample was ground and then heated to 550 °C (heating rate of 5 °C /min) for 4 h in a muffle furnace. The obtained sample was a kaolin/g-C_3N_4 composite.

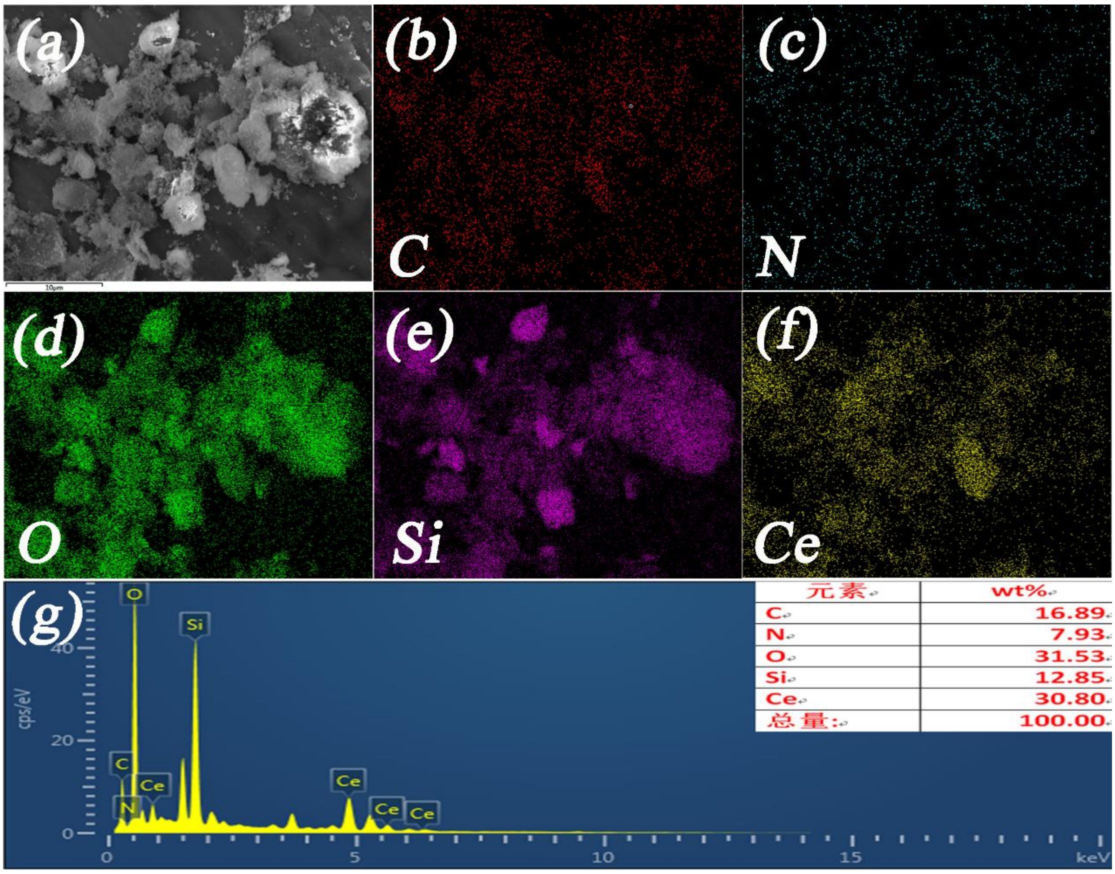

Figure S1. (**b**–**f**) element mapping of C, N, O, Si, and Ce for kaolin/CeO_2/g-C_3N_4composite, the upper left corner (**a**) shows the corresponding images. (**g**) EDS spectrum.

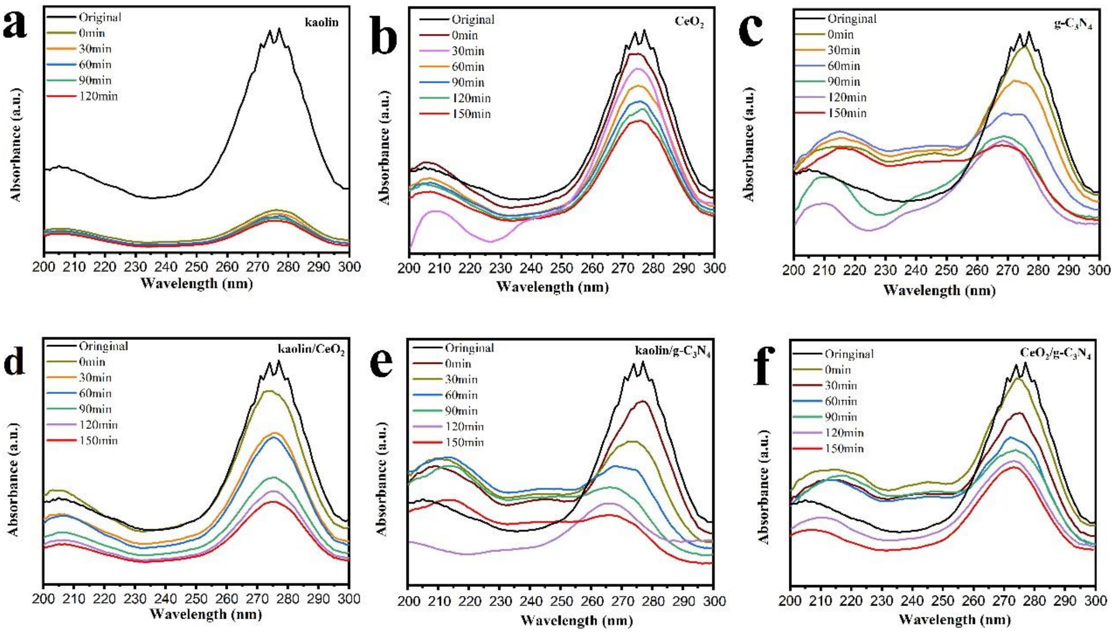

Figure S2. The changes in UV-vis spectra of CIP in liquid phase over kaolin (**a**), CeO_2 (**b**), g-C_3N_4 (**c**), kaolin/CeO_2 (**d**), kaolin/g-C_3N_4 (**e**) and CeO_2/g-C_3N_4 (**f**) composite vs irradiation time.

© 2020 by the authors. Submitted for possible open access publication under the terms and conditions of the Creative Commons Attribution (CC BY) license (http://creativecommons.org/licenses/by/4.0/).
